# Mammography Among Women Residing in Urban Versus Rural Utah: Breast Cancer Survival

**DOI:** 10.1002/cam4.70505

**Published:** 2024-12-16

**Authors:** Quinn Tanner, Chun‐Pin Chang, Karen Curtin, James VanDerslice, Lisa Gren, Vikrant Deshmukh, Michael Newman, Ankita Date, Mark Dodson, N. Lynn Henry, Mia Hashibe

**Affiliations:** ^1^ Division of Public Health, Department of Family & Preventive Medicine University of Utah School of Medicine Salt Lake City Utah USA; ^2^ Division of Public Health, Department of Family and Preventive Medicine Huntsman Cancer Institute, University of Utah School of Medicine Salt Lake City Utah USA; ^3^ Department of Internal Medicine University of Utah School of Medicine Salt Lake City Utah USA; ^4^ Pedigree and Population Resource, Huntsman Cancer Institute University of Utah School of Medicine Salt Lake City Utah USA; ^5^ University of Utah Health Salt Lake City Utah USA; ^6^ Pedigree and Population Resource, Population Sciences Huntsman Cancer Institute Salt Lake City Utah USA; ^7^ Gynecologic Oncology Intermountain Healthcare Salt Lake City Utah USA; ^8^ Division of Hematology/Oncology, Department of Internal Medicine University of Michigan Medical School Ann Arbor Michigan USA

## Abstract

**Background:**

Annual or biennial breast cancer screenings are recommended for women 40 and older. Women residing in rural areas have worse breast cancer survival rates than urban women, but no study has focused on rural versus urban residence in Utah regarding breast cancer screening and mortality.

**Methods:**

Cases (*n* = 14,516) were women aged > 39 diagnosed with a first primary invasive breast cancer between 1998 and 2017 in Utah. Controls (*n* = 63,117) without a history of breast cancer were matched to cases by birth year and birth state. Mammography screening status was identified by Current Procedural Terminology (CPT) codes. Logistic regression was used to assess the odds of breast cancer diagnosis. The Cox proportional hazards model was used to assess survival outcomes for rural and urban breast cancer patients based on screening status.

**Results:**

Screening mammography usage among rural patients diagnosed with breast cancer was lower (17.7%) than urban usage (20.7%). Usage of screening mammograms resulted in higher odds of breast cancer diagnosis at localized stage rather than at a regional and distant stage. Rural breast cancer cases had a higher proportion of deaths, and a lower proportion screened, than urban breast cancer cases. Hazard ratios showed that screening mammography usage was associated with better survival among both rural (HR = 0.50, 95% CI = 0.44–0.57) and urban (HR = 0.56, 95% CI = 0.39–0.82) breast cancer cases.

**Conclusion:**

Screening mammography usage was associated with better overall survival regardless of place of residence. Removing barriers and improving information regarding breast cancer screenings are needed in both rural and urban settings in Utah to increase mammography usage, with the overall goal of increasing early detection and outcomes of breast cancer.

## 1 Introduction

Female breast cancer is the most commonly diagnosed cancer and the leading cause of cancer death worldwide among women [1, 2, 3]. Breast cancer screening mammography guidelines have varied slightly over time and among agencies, but generally major institutions—including the United States Preventive Services Taskforce (USPSTF), the American Cancer Society (ACS), and the American College of Radiology (ACR)—recommend women at average risk of developing breast cancer undergo screening annually or biennially starting between ages 40 and 50 [1, 4, 5, 6, 7, 8, 9]. Early detection of breast cancer from a screening mammogram is associated with a reduced risk of breast cancer death by 20%–40% [7].

Overall breast cancer incidence in the United States has increased over the past 30 years. Due to advances in treatment and increased utilization of breast cancer screening, the overall death rate has dropped by 12.5/100,000 since 1991 [10, 11]. However, recent studies have shown disparities in breast cancer screening mammography usage and survival outcomes between urban and rural populations. Some national and statewide studies have found that rural women are less likely to have undergone screening mammography [12, 13, 14, 15, 16], were more likely to have their breast cancer diagnosed at later stages [12, 17, 18, 19], and had worse survival outcomes [20] than their urban counterparts. Few studies have investigated the difference in screening mammography usage among rural versus urban women in Utah specifically [9, 21], and one of these two studies included other states in its investigation and did not focus on Utah exclusively [9]. No studies have investigated both screening mammography usage and survival outcomes between these populations.

Further analysis is required to assess differences in rural versus urban women for screening mammography use and breast cancer survival outcomes in more areas of the country. This is particularly true for the state of Utah, where 25 of Utah's 29 counties qualify as “rural” or “frontier” [22]. Utah is below the national average for screening mammography usage [21], with 62.7% of Utah women reporting having been screened in 2020 versus 69.0% nationally [23]. Rural Utah women were found to have lower and later usage of screening mammograms than their urban counterparts [9].

A study that investigates rural versus urban disparities in Utah is not only necessary to address this knowledge gap in how rurality affects breast cancer screening, diagnosis, and outcomes in Utah—but performing a study based in Utah also provides access to linked secondary datasets that include decades worth of demographic and medical information for the state's population. The large, historical, and data‐rich population in these Utah‐based linked datasets were valuable resources in addressing our primary aims. These aims were to (1) identify breast cancer screening mammography usage among Utah women using linked demographic and cancer databases, (2) assess whether screening mammography usage differed between rural and urban Utah women diagnosed with breast cancer, and (3) assess differences in survival outcomes between rural and urban Utah women diagnosed with breast cancer based on their screening status. Our a priori hypotheses were that rural Utah women with breast cancer would have lower screening mammography usage than urban Utah women with breast cancer, and that survival outcomes would be worse for rural Utah women with breast cancer than urban Utah women with breast cancer.

## 2 Methods

### 2.1 Data Sources

This study examined (1) breast cancer mammography usage among breast cancer cases and their matched controls, including examining demographic factors; and (2) breast cancer mammography usage among urban versus rural breast cancer cases, including examining demographic factors, clinical factors, and survival outcomes. Data were drawn from the Utah Population Database (UPDB), which links data from the Utah Cancer Registry (UCR), statewide healthcare facility data (ambulatory surgery, emergency department, and inpatient hospital claims), and electronic medical records from the two biggest medical care providers in the state: University of Utah Health system and Intermountain Healthcare. This study was approved by the University of Utah Institutional Review Board as well as the Resource for Genetic and Epidemiologic Research (the UPDB regulatory body).

### 2.2 Study Population

Women with breast cancer were identified using diagnosis data from the UCR. We included female breast cancer patients meeting the following eligibility criteria: (1) diagnosed between 1998 and 2017, (2) residential address in Utah at time of diagnosis, (3) first primary cancer diagnosis, (4) age at diagnosis was 40 or older, (5) at least 3 years of recorded medical history in the linked databases before diagnosis date, and (6) cancer stage was not in situ or unknown. Women under the age of 40 were excluded because breast cancer screening recommendations do not recommend annual or biennial screenings for women at average risk of breast cancer until after age 40. We excluded individuals with less than 3 years of recorded medical history in order to remove those who were entered into the medical system to seek treatment or diagnosis in response to early symptoms of breast cancer.

Women without breast cancer from the Utah general population were matched to cases at up to a 5:1 proportion on birth year and birth state. There were 14,516 cases and 63,117 controls included in analysis (total *n* = 77,633). The index date was defined for all study participants in order to calculate time to event in our survival analysis. Index date was the date of breast cancer diagnosis for cases and for controls was the diagnosis date of the case they were matched to.

### 2.3 Study Variables

An individual was defined as receiving a screening mammogram if they had any screening codes in their medical history between 1 and 3 years prior to the index date. Screening mammography codes were derived from International Classification of Diseases (ICD), Current Procedural Terminology (CPT), and Healthcare Common Procedure Coding System (HCPCS) codes (Table S1). Screening mammography codes were differentiated from diagnostic mammography codes in order to focus on use of recommended breast cancer screening practices rather than tests done as a result of breast cancer symptoms. A cutoff 1 year prior to the index date was used to ensure that diagnostic mammograms were not miscategorized as screening mammograms. Where there were multiple body mass index (BMI) records in an individual's driver license records, the most recent BMI recorded at least 1 year prior to the index date was used in analysis. For the 6.7% of the study population who were missing BMI data, we imputed BMI using multiple imputation with linear regression, with age at index date, sex, race, and Charlson Comorbidity Index (CCI) score [24]. CCI scores were calculated based on patients' records before the index date.

Rural versus urban status was only available for the breast cancer cases since it was drawn from the UCR database. Among cases, rural versus urban status was assigned using census‐tract residential data and categorizing it according to standards from the United States Department of Agriculture's (USDA) Rural Urban Commuting Area (RUCA) classification system, Categorization C (urban cases were in RUCA codes 1, 1.1, 2, 2.1, 3, 4.1, 5.1, 7.1, 8.1, and 10.1; rural cases were in RUCA codes 4, 4.2, 5, 5.2, 6, 6.1, 7, 7.2, 7.3, 7.4, 8, 8.2, 8.3, 8.4, 9, 9.1, 9.2, 10, 10.2, 10.3, 10.4, 10.5, 10.6). Of the 14,516 breast cancer cases, two were missing RUCA information, 1652 (11%) were identified as rural, and 12,862 (89%) were identified as urban.

### 2.4 Statistical Analysis

All analyses were conducted using SAS 9.4 (SAS Institute Inc., Cary, NC). Two‐sided Pearson's chi‐square tests were used to assess differences in the demographic characteristics of breast cancer cases versus controls. Demographic and clinical characteristics were also assessed among rural versus urban breast cancer cases using two‐sided Pearson's chi‐squared tests, and stratified based on urban versus rural residential status. Conditional logistic regression was used to calculate odds ratios (ORs) for the odds of breast cancer diagnosis given screening mammography usage adjusted for race, ethnicity, BMI, education, and CCI score.

Cox proportional hazards models were used to calculate hazard ratios (HRs) for the risk of death between rural and urban breast cancer cases. Time to event was calculated as the time from cancer diagnosis to death, lost‐to‐follow‐up, or the end of the study. Potentially confounding factors that were adjusted on for the survival analysis included age at diagnosis, diagnosis year, race, ethnicity, BMI, education, and CCI—as these meet the three properties of confounders that (1) the covariate is a risk factor for death; (2) the covariate is associated with the exposure; and (3) the covariate is not a mediator.

The proportional hazards assumption was tested for each Cox proportional hazards model by including interactions between the predictors and time in the model. For models where the assumption was violated, we compared the estimates from the flexible parametric survival model with restricted cubic splines [25, 26] and the Cox model. The estimate from the flexible model was used if the inference was different between the two models.

### 2.5 Ethical Approval

This study was approved by the University of Utah Institutional Review Board (IRB 00099680), as well as the Resource for Genetic and Epidemiologic Research (the UPDB regulatory body). The IRB granted an exemption for written informed consent for participants. Linked databases have strict data sharing and participant anonymity procedures to ensure patient confidentiality. Any patient who indicated they did not want to be included in scientific study analysis was removed from the dataset. Names and other information that could personally identify any patient were removed from data by the data registries before being distributed to IRB‐approved reviewers.

## 3 Results

Breast cancer cases were significantly more likely to be a racial or ethnic minority (*p* < 0.001), to have less education (*p* < 0.001), to be overweight or obese (*p* < 0.001), to have a first‐degree family member with a history of breast cancer (*p* < 0.001), to be a non‐smoker (*p* = 0.031), to have a CCI score of 0 (*p* = 0.004), and to have died during the study period (*p* < 0.001) than controls (Table 1). Note that for all tables, statistically significant p‐values at the 0.05 threshold, along with odds ratios and hazard ratios whose 95% confidence intervals did not include 1, are formatted in bold.

**TABLE 1:** Distribution of demographic characteristics of cases versus controls.

Characteristic	Cases versus Controls		
	Cases, *n* (%)	Controls, *n* (%)	*p*‐value
Total, *n*	14,516	63,117	
Age (years)			
Mean (SD)	62.44 (12.66)	62.63 (12.71)	0.553
40–49	2615 (18.0)	11,223 (17.8)	
50–59	3727 (25.7)	15,951 (25.3)	
60–69	3883 (26.8)	16,861 (26.7)	
70–79	2731 (18.8)	12,087 (19.2)	
80–101	1560 (10.8)	6995 (11.1)	
Race and ethnicity			
White, non‐Hispanic	12,610 (86.9)	55,597 (88.1)	**< 0.001**
Hispanic	1393 (9.6)	4981 (7.9)	
Asian, non‐Hispanic	139 (1.0)	460 (0.7)	
Native Hawaiian or other Pacific Islander, non‐Hispanic	45 (0.3)	104 (0.2)	
Black or African American, non‐Hispanic	—	—	
Native American or Alaska Native, non‐Hispanic	—	—	
Multiple races	281 (1.9)	1008 (1.6)	
Unknown	—	—	
Education level			
Some high school or less	1405 (9.7)	6705 (10.6)	**< 0.001**
High school degree	3384 (23.3)	16,823 (26.7)	
Some college	3202 (22.1)	15,774 (25.0)	
College degree	1458 (10.0)	7038 (11.2)	
Post college	1028 (7.1)	4227 (6.7)	
Unknown	4039 (27.8)	12,550 (19.9)	
BMI^a^			
Underweight (< 18.5)	235 (1.6)	1157 (1.8)	**< 0.001**
Normal (18.5–24.9)	5979 (41.2)	27,936 (44.3)	
Overweight (25.0–29.9)	4781 (32.9)	19,884 (31.5)	
Obese (≥ 30)	3521 (24.3)	14,140 (22.4)	
Family history of breast cancer in first‐degree relative			
Yes	2149 (14.8)	6796 (10.8)	**< 0.001**
No	12,367 (85.2)	56,321 (89.2)	
Smoking status			
Smoker	1289 (8.9)	5970 (9.5)	**0.031**
Non‐smoker	13,227 (91.1)	57,147 (90.5)	
Vital status			
Alive	10,807 (74.5)	53,733 (85.1)	**< 0.001**
Deceased	3709 (25.6)	9384 (14.9)	
Charlson Comorbidity Index (CCI)			
0	7341 (50.6)	31,113 (49.3)	**0.004**
1	3585 (24.7)	15,602 (24.7)	
2+	3590 (24.7)	16,402 (26.0)	

*Note:* Statistically significant *p*‐values at the 0.05 threshold, along with odds ratios and hazard ratios whose 95% confidence intervals did not include 1, are formatted in bold.

^a^ BMI includes imputed BMI for missing data or for all participants or BMIs recorded less than 1 year before diagnosis date for cases, original data includes *n* = 5174 missing responses among cases and controls (6.7%).

A higher proportion of breast cancer cases (20.4%) than controls (19.4%) had received a screening mammogram (Table 2). Conditional logistic regression was used to adjust on age and birth state, and after further adjusting with race, smoking status, first‐degree family member with breast cancer history, ethnicity, education level, BMI, and CCI score, women who underwent a screening mammogram had 1.15 times higher odds of being diagnosed with breast cancer compared to non‐users (OR = 1.15, 95% CI = 1.09–1.22) (Table 2). However, the increased odds of a breast cancer diagnosis was only at the localized stage. The odds of a breast cancer diagnosis for women in the regional or distant cancer stages were 20% lower among those who underwent screening mammography compared to those who did not (OR = 0.80, 95% CI = 0.73–0.87).

**TABLE 2:** Adjusted odds ratio of breast cancer given a screening mammogram was administered between 1 and 3 years before index date (*n* = 77,633).

	Cases, *n* (%)	Controls, *n* (%)	Odds ratio (95% CI)^a^
Cases with *all cancer stages*, and their matched controls			
Mammogram performed in timeframe:			
No mammogram	11,559 (79.6)	50,858 (80.6)	Reference
Mammogram	2957 (20.4)	12,259 (19.4)	**1.15 (1.09–1.22)**
Number of mammograms performed in timeframe			
0	11,559 (79.6)	50,858 (80.6)	Reference
1	1537 (10.6)	7333 (11.6)	1.00 (0.94–1.06)
2	1215 (8.4)	4345 (6.9)	**1.38 (1.28–1.48)**
3+	205 (1.4)	581 (0.9)	**1.67 (1.40–2.01)**
Cases with *local cancer stages only*, and their matched controls			
Mammogram performed in timeframe:			
No mammogram	6917 (76.3)	31,647 (80.2)	Reference
Mammogram	2149 (23.7)	7821 (19.8)	**1.40 (1.32–1.49)**
Number of mammograms performed in timeframe:			
0	6917 (76.3)	31,647 (80.2)	Reference
1	1054 (11.6)	4650 (11.8)	**1.14 (1.06–1.24)**
2	934 (10.3)	2785 (7.1)	**1.77 (1.62–1.93)**
3+	161 (1.8)	386 (1.0)	**2.14 (1.73–2.65)**
Cases with *regional and distant cancer stages only*, and their matched controls			
Mammogram performed in timeframe:			
No mammogram	4642 (85.2)	19,211 (81.2)	Reference
Mammogram	808 (14.8)	4438 (18.8)	**0.80 (0.73–0.87)**
Number of mammograms performed in timeframe:			
0	4642 (85.2)	19,211 (81.2)	Reference
1	483 (8.9)	2683 (11.4)	**0.79 (0.70–0.87)**
2	281 (5.2)	1560 (6.6)	**0.80 (0.70–0.92)**
3+	44 (0.8)	195 (0.8)	0.91 (0.63–1.31)

*Note:* Statistically significant *p*‐values at the 0.05 threshold, along with odds ratios and hazard ratios whose 95% confidence intervals did not include 1, are formatted in bold.

^a^ Conditional logistic regression was used to adjust on age and birth state. The model additionally adjusted on race, smoking status, first‐degree family member with breast cancer history, ethnicity, education level, BMI, and CCI score.

Demographic characteristics of rural versus urban breast cancer cases show that rural breast cancer cases were significantly more likely than urban breast cancer cases to be non‐Hispanic White (*p* < 0.001), to have a high school education or less (*p* < 0.001), to have a CCI score of 2+ (*p* = 0.040), and to have died or been lost to follow up within 5 years post‐diagnosis (rural cases with less than 5 years of follow‐up = 40.7%, urban cases with less than 5 years of follow‐up = 39.3%). There was no statistically significant difference in age, BMI, having a first‐degree family member with breast cancer, or smoking status between rural and urban breast cancer patients (Table 3).

**TABLE 3:** Distribution of demographic characteristics of urban versus rural cases.

Characteristic	Rural cases versus urban cases^a^		
	Urban, *n* (%)	Rural, *n* (%)	*p*‐value
Total, *n*	12,862	1652	
Age (years)			
Mean (SD)	62.34 (12.67)	63.17 (12.53)	0.073
40–49	2339 (18.2)	276 (16.7)	
50–59	3318 (25.8)	409 (24.8)	
60–69	3448 (26.8)	434 (26.3)	
70–79	2379 (18.5)	351 (21.3)	
80–101	1378 (10.7)	182 (11.0)	
Race and ethnicity			
White, non‐Hispanic	11,068 (86.1)	1541 (93.3)	**< 0.001**
Hispanic	1318 (10.3)	74 (4.5)	
Asian, non‐Hispanic	—	—	
Native Hawaiian or other Pacific Islander, non‐Hispanic	—	—	
Black or African American, non‐Hispanic	—	—	
Native American or Alaska native, non‐Hispanic	—	—	
Multiple races	255 (2.0)	26 (1.6)	
Unknown	—	—	
Education level			
Some high school or less	1195 (9.3)	210 (12.7)	**< 0.001**
High school degree	2981 (23.2)	402 (24.3)	
Some college	2857 (22.2)	345 (20.9)	
College degree	1323 (10.3)	135 (8.2)	
Post college	945 (7.4)	83 (5.0)	
Unknown	3561 (27.7)	477 (28.9)	
BMI^b^			
Underweight (< 18.5)	207 (1.6)	28 (1.7)	0.091
Normal (18.5–24.9)	5307 (41.3)	670 (40.6)	
Overweight (25.0–29.9)	4267 (33.2)	514 (31.1)	
Obese (≥ 30)	3081 (24.0)	440 (26.6)	
Family history of breast cancer in first‐degree relative			
Yes	1918 (14.9)	231 (14.0)	0.317
No	10,944 (85.1)	1421 (86.0)	
Smoking status			
Smoker	1144 (8.9)	145 (8.8)	0.875
Non‐smoker	11,718 (91.1)	1507 (91.2)	
Vital status			
Alive	9620 (74.8)	1185 (71.7)	**0.007**
Deceased	3242 (25.2)	467 (28.3)	
Follow‐up time (years)			
Mean (SD)	7.27 (4.68)	7.14 (4.70)	**< 0.001**
< 5	5051 (39.3)	672 (40.7)	
5–9	4234 (32.9)	544 (32.9)	
10–14	2523 (19.6)	304 (18.4)	
15–19	1054 (8.2)	132 (8.0)	
Charlson Comorbidity Index (CCI)			
0	6515 (50.7)	825 (49.9)	**0.040**
1	3205 (24.9)	380 (23.0)	
2+	3142 (24.4)	447 (27.1)	

*Note:* Statistically significant *p*‐values at the 0.05 threshold, along with odds ratios and hazard ratios whose 95% confidence intervals did not include 1, are formatted in bold.

^a^ RUCA status was not available for two cases; cases without RUCA data were not included in this table.

^b^ BMI includes imputed BMI for missing data or for all participants or BMIs recorded less than 1 year before diagnosis date for cases, original data include *n* = 5174 missing responses among cases and controls (6.7%).

Clinical characteristics of rural breast cancer cases differed significantly compared to urban breast cancer cases: rural breast cancer cases were less likely than urban breast cancer cases to have received radiation as part of first‐course treatment (*p* < 0.001), and rural breast cancer cases had a lower proportion (3.0%) of screening mammogram (*n* = 292, 17.7%) than urban breast cancer cases (*n* = 2664, 20.7%) (Table 4). Rural and urban breast cancer cases did not significantly differ in cancer stage at diagnosis, first‐course treatment combination, or other specific treatments that were used during first‐course treatment. Breast cancer cases who received a mastectomy were similar between rural and urban groups, though there were differences between rural and urban cases in terms of type of mastectomy received. Urban cases were more likely than rural cases to have received a partial mastectomy (56.2% of urban cases vs 51.1% of rural cases). Rural cases were more likely than urban cases to have had a modified radical mastectomy (28.15% of rural cases versus 23.68% of urban cases). Urban and rural cases did not differ or differed by less than a percentage point for extended radical mastectomy, subcutaneous mastectomy, total simple mastectomy, radical mastectomy NOS, surgery NOS, local tumor destruction NOS, mastectomy NOS, no mastectomy, or unknown mastectomy status (data not shown).

**TABLE 4:** Clinical characteristics of breast cancer cases in Utah between 1998 and 2017, stratified by residential status.

Characteristic	Total (%)^a^	Urban, *n* (%)	Rural, *n* (%)	*p*‐value
Total, *n*	14,514	12,862	1652	
Cancer stage				
Localized	9064 (62.5)	8014 (62.3)	1050 (63.6)	0.187
Regional	4779 (32.9)	4263 (33.1)	516 (31.2)	
Distant	671 (4.6)	585 (4.6)	86 (5.2)	
Screening mammography between 1 and 3 years before diagnosis				
Yes	2956 (20.4)	2664 (20.7)	292 (17.7)	**0.004**
No	11,558 (79.6)	10,198 (79.3)	1360 (82.3)	
Received surgery as first‐course treatment	13,806 (95.1)	12,236 (95.1)	1570 (95.0)	0.864
Received radiation as first‐course treatment	8181 (56.4)	7317 (56.9)	864 (52.3)	**< 0.001**
Received endocrine therapy as first‐course treatment	6566 (45.2)	5806 (45.1)	760 (46.0)	0.507
Received chemotherapy as first‐course treatment	5814 (40.1)	5174 (40.2)	640 (38.7)	0.246

*Note:* Statistically significant *p*‐values at the 0.05 threshold, along with odds ratios and hazard ratios whose 95% confidence intervals did not include 1, are formatted in bold.

^a^ RUCA status was not available for two cases; cases without RUCA data were not included in this table.

Both rural (HR = 0.56, 95% CI = 0.39–0.82) and urban (HR = 0.50, 95% CI = 0.44–0.57) breast cancer cases who had at least one screening mammogram within the specified timeframe had a lower risk of death than those who had no screening mammogram (Table 5).

**TABLE 5:** Risk of death among breast cancer cases based on the number of screening mammograms recorded between 1 and 3 years before diagnosis date, stratified by residential status (*n* = 14,514).

	Urban			Rural		
	Living, *n* (%)	Deceased, *n* (%)	Hazard ratio (95% CI)^a^	Living, *n* (%)	Deceased, *n* (%)	Hazard ratio (95% CI)^a^
Mammogram performed						
No mammogram	7249 (75.4)	2949 (91.0)	Reference	926 (78.1)	434 (92.9)	Reference
Mammogram	2371 (24.7)	293 (9.0)	**0.50 (0.44–0.57)**	259 (21.9)	33 (7.1)	**0.56 (0.39–0.82)**
Number of mammograms						
0	7249 (75.4)	2949 (91.0)	Reference	926 (78.1)	434 (92.9)	Reference
1	1217 (12.7)	165 (5.1)	**0.54 (0.46–0.63)**	127 (10.7)	28 (6.0)	0.85 (0.57–1.26)
2	—	—	—	—	—	—
3+	—	—	—	—	—	—

*Note:* Statistically significant *p*‐values at the 0.05 threshold, along with odds ratios and hazard ratios whose 95% confidence intervals did not include 1, are formatted in bold.

^a^ Adjusted on age, diagnosis year, race, ethnicity, education level, BMI, and CCI score.

## 4 Discussion

Usage of screening mammograms resulted in higher odds of breast cancer diagnosis at a localized stage, rather than at a regional or distant stage. This indicates that although usage of screening mammograms results in a greater number of breast cancer diagnoses, these breast cancer cases are caught at earlier stages—which is ultimately the goal of cancer screening. In our study, among Utah women who were diagnosed with breast cancer, screening mammography usage resulted in significantly better survival outcomes. Of these breast cancer cases, rural breast cancer patients had 3.0% lower screening mammography usage and 3.1% more deaths in the study timeframe compared to urban breast cancer patients. First‐course treatment did not differ between urban breast cancer cases and rural breast cancer cases significantly except for radiation use, where urban cases were 4.6% more likely to have received radiation as part of their first‐course treatment than rural cases. Finally, rural breast cancer cases had a slightly higher proportion who died from all causes during the study period than urban breast cancer cases.

These findings expand on knowledge gained from two other studies that included Utah and examined differences between rural and urban women's screening mammography usage: Schootman et al.'s 2003 analysis and Henry et al.'s 2010 analysis [9, 21]. The 2003 study by Schootman et al. used Surveillance, Epidemiology, and End Results (SEER) data to track breast cancer cases of ductal carcinoma in situ (DCIS) between 1973 and 1997 as a proxy for nonadherence to screening mammography recommendations [9]. Their findings suggested that there were “differences in utilization of mammography between both populations; the increase [in mammography usage] was lower and started later for rural women.” The 2014 study by Henry et al. used data from the 2010 Utah Behavioral Risk Factor Surveillance System (BRFSS) to assess the odds of women not receiving a screening mammogram in the prior 2 years in relation to their travel time to the nearest mammography facility, geographic accessibility, and rural versus urban status [21]. Unlike our own findings and the findings from the SEER study, the BRFSS study did not find a significant correlation between these geographic markers and breast cancer screening utilization. Instead, they found that more significant indicators of “nonadherence to mammography screening guidelines in Utah” included not having a regular physician, lacking health insurance, being aged 40–49 (rather than aged 50–74), having income less than $25,000, and the presence of three or more children in the house.

Our study also investigated survival outcomes in association with screening mammography usage, showing an association between utilizing these screenings and lower risk of death. Though the findings from the BRFSS study did not find differences in breast cancer screening usage between rural and urban Utah women, the investigators noted that study limitations may have caused their results to be biased toward the null ‐ including stratifying their rural versus urban population into three age groups (40–50, 51–74, and 75+). Though their findings when stratified by age lacked statistical significance, the odds of rural women in all age stratifications of having “nonadherence” to recommended screening mammography usage were higher than for urban women [21], which aligns with our results. Additionally, their focus on other demographic factors that did result in statistical significance could inform future studies investigating interventions to reduce screening mammography disparities.

We found that rural Utah women had lower screening mammography use than urban Utah women. Rural Utah women may not be using screening mammograms as frequently as urban Utah women for a number of reasons, including noted disparities in access to care between rural and urban individuals. Lower screening rates among rural Utah women compared to urban Utah women may be a marker for differences in access to healthcare in general, resulting in the differences seen in odds of death from all causes within the study period. Disparities in access to healthcare may include those noted in the BRFSS study, such as irregular access to a primary care provider, lower health insurance rates, lower annual income, and household demographics. Other potential screening mammography disparities that warrant further study include increased distance to screening providers and cancer care centers, and less access to information regarding recommended screening practices.

There were some limitations in this study. Utilizing medical codes to identify screening mammograms offered some strengths to the study design, but also resulted in lower‐than‐expected screening mammography usage among our study population. While the self‐reported screening mammography usage among Utah women in the BRFSS results were between 60% and 70%, our results had only 20.6% of our total population that were coded as having a screening mammogram in the study timeframe. We may have under‐captured the true number of Utah women over 40 who received a screening mammogram. This could be because mammograms can be performed at many different types of facilities, including imaging centers, mobile health units, and doctors' offices. It is possible that the linked data and mammography codes from the two largest medical care providers did not capture a large portion of mammograms performed at these other facilities in the state. Another possible reason the reported number of mammograms was lower than other reports could be because we reported on screening mammography codes between 1 and 3 years before the index date rather than the more commonly used window of 2 years prior to cancer diagnosis date. However, underreporting of these codes would bias results toward the null, and there is no indication that reporting accuracy varied between subgroups (including cases vs controls, or urban cases vs rural cases). Additionally, though a large geographic area of Utah is rural, a relatively small proportion of the population diagnosed with breast cancer live in these areas, resulting in limited statistical power in some analyses. However, the overall study population was large (*n* = 77,633) and offered more than enough power to study associations outlined in our primary aims. We also assumed that rural residential status at time of cancer diagnosis indicated a history of rural residency and did not take into account possible relocations. Finally, these findings may not be generalizable to other areas of the United States due to differences in demographic characteristics.

This study also had several unique strengths. The large, linked databases allowed us to capture a study population of nearly 80,000 Utah women. Secondary data analysis utilizing healthcare data also decreased the possibility of recall bias that would have been present as a result of a study based on surveys or interviews, including baseline data for participants' CCI scores, and 93.3% of participants' pre‐index‐date BMI. With data from the UCR, we also were able to access first‐course cancer treatments and cancer stages upon diagnosis. Unlike prior cited studies [9, 21], our investigation used medical codes rather than proxies or self‐reported survey results to determine if Utah women had received a mammogram. Beyond limiting recall or other biases among our study population, these codes also differentiated between screening mammograms and diagnostic mammograms, allowing our study to focus on use of recommended breast cancer screening practices rather than tests done as a result of breast cancer symptoms or to evaluate concerning findings on screening mammograms. Our study also captured data from years ranging from 2008 to 2017, adding more up‐to‐date and modern demographic information to our analyses. Our sensitivity analyses showed that screening mammography codes occurred more frequently for the entire study population after 2012 (11.8%) compared to prior to 2013 (32.6%), indicating that using screening mammography codes in analyses will continue to offer useful insights as the years progress and these codes are used with more frequency.

### 4.1 Conclusion

Screening mammography usage was associated with decreased risk of regional or distant breast cancer diagnoses. Mammography use only differed slightly between rural and urban Utah women over 40 who were diagnosed with breast cancer. However, screening rates among both rural and urban study populations were lower than the national average. Further research should focus on investigating the reasons for the state's relatively low screening rates for all populations, including barriers to screening services. These may include distance to treatment centers and health insurance status. This knowledge is key to identify and implement successful outreach efforts to increase screenings among all populations.

## Author Contributions

**Quinn Tanner:** formal analysis (equal), investigation (equal), methodology (equal), project administration (lead), resources (lead), validation (supporting), writing – original draft (lead), writing – review and editing (lead). **Chun‐Pin Chang:** conceptualization (equal), data curation (lead), formal analysis (equal), funding acquisition (supporting), investigation (equal), methodology (equal), project administration (supporting), resources (supporting), software (supporting), supervision (lead), validation (lead), visualization (supporting), writing – original draft (equal), writing – review and editing (equal). **Karen Curtin:** data curation (supporting), funding acquisition (supporting), resources (supporting), writing – review and editing (supporting). **James VanDerslice:** validation (supporting), visualization (equal), writing – review and editing (equal). **Lisa Gren:** formal analysis (supporting), methodology (supporting), writing – review and editing (equal). **Vikrant Deshmukh:** writing – review and editing (supporting). **Michael Newman:** data curation (supporting), funding acquisition (supporting), writing – review and editing (supporting). **Ankita Date:** writing – review and editing (supporting). **Mark Dodson:** writing – review and editing (supporting). **N. Lynn Henry:** writing – review and editing (equal). **Mia Hashibe:** conceptualization (lead), data curation (supporting), formal analysis (supporting), funding acquisition (lead), investigation (supporting), methodology (supporting), project administration (supporting), resources (supporting), software (supporting), supervision (supporting), validation (supporting), visualization (supporting), writing – original draft (supporting), writing – review and editing (supporting).

## Conflicts of Interest

The authors declare no conflicts of interest.

## Supporting information


**Table S1.** Screening mammography codes.
**Table S2.** Diagnostic mammography codes.
**Table S3.** Risk of death among breast cancer cases based on whether a screening mammogram was performed between 1 and 3 years before diagnosis date, stratified by residential status (*n* = 14,514).
**Table S4.** Adjusted odds ratio of breast cancer given a screening mammogram was administered between 1 and 3 years before index date, stratified by the index date year (*n* = 77,633).

## Data Availability

The data that support the findings of this study are available upon request with appropriate approval from the UPDB oversight committee, the Resource for Genetic and Epidemiologic Research (RGE), and IRB approval.
