# Supplementary material for: Mammography Among Women Residing in Urban Versus Rural Utah: Breast Cancer Survival
Source: Cancer Med. 2024 Dec 16;13(24):e70505. doi: 10.1002/cam4.70505 (PMC11648033; doi:10.1002/cam4.70505)
Supplement: Supplementary file 1 — Table S1. Screening mammography codes. Table S2. Diagnostic mammography codes. Table S3. Risk of death among breast cancer cases based on whether a screening mammogram was performed between 1 and 3 years before diagnosis date, stratified by residential status (n = 14,514). Table S4. Adjusted odds ratio of breast cancer given a screening mammogram was administered between 1 and 3 years before index date, stratified by the index date year (n = 77,633). [file CAM4-13-e70505-s001.docx]

| **Supplemental Table 1. Screening Mammography Codes** | | |
| --- | --- | --- |
| Code Source | Code | Description |
| HCPCS | G0202 | Screening mammography, producing direct digital image, bilateral, all views. |
| CPT | 76092 | Screening mammography, bilateral (two view film study of each breast). |
| CPT | 77057 | Screening mammography, bilateral (two view film study of each breast). |
| CPT | 77063 | Screening digital breast tomosynthesis, bilateral. |
| CPT | 77067 | Screening mammography, bilateral (2-view study of each breast), including CAD when performed. |
| ICD-9-CM | V76.11 | Screening mammogram for high-risk patient. |
| ICD-10-CM | Z12.31 | Screening mammogram for high-risk patient. |

| **Supplemental Table 2. Diagnostic Mammography Codes** | | |
| --- | --- | --- |
| Code Source | Code | Description |
| HCPCS | G0204 | Diagnostic mammography, direct digital image, bilateral, all views. |
| HCPCS | G0205 | Diagnostic mammography, film processed to produce digital image analyzed for potential abnormalities, bilateral, all views. |
| HCPCS | G0206 | Diagnostic mammography, including CAD when performed, unilateral. |
| HCPCS | G0207 | Diagnostic mammography, producing direct digital image, unilateral, all views. |
| HCPCS | G0279 | Diagnostic digital breast tomosynthesis, unilateral or bilateral. |
| CPT | 76090 | Diagnostic mammography, unilateral. |
| CPT | 76091 | Diagnostic mammography, bilateral. |
| CPT | 77065 | Diagnostic mammography, including CAD when performed; unilateral. |
| CPT | 77066 | Diagnostic mammography, including CAD when performed, bilateral. |

| **Supplemental Table 3: Risk of death among breast cancer cases based on whether a screening mammogram was performed between 1 year and 3 years before diagnosis date, stratified by residential status (n = 14,514)** | | | | | | |
| --- | --- | --- | --- | --- | --- | --- |
|  | Urban | | | Rural | | |
|  | Living, n (%) | Deceased, n (%) | Hazard Ratio (95% CI)* | Living, n (%) | Deceased, n (%) | Hazard Ratio (95% CI)* |
| First Degree Family Member with Breast Cancer** | | | | | | |
| No First-Degree Family Member Diagnosed with Breast Cancer (n = 12,365) | | | | | | |
| No mammogram | 6,301 (75.8%) | 2,380 (90.6%) | *Reference* | 802 (77.6%) | 360 (92.8%) | *Reference* |
| Mammogram | 2,015 (24.2%) | 248 (9.4%) | **0.55 (0.48,0.63)** | 231 (22.4%) | 28 (7.2%) | 0.70 (0.47,1.04) |
| At Least One First-Degree Family Member Diagnosed with Breast Cancer (n = 2,149) | | | | | | |
| No mammogram | 948 (72.7%) | 569 (92.7%) | *Reference* | - | - | *Reference* |
| Mammogram | 356 (27.3%) | 45 (7.3%) | 0.98 (0.78,1.22) | - | - | - |
| Cancer Stage | | | | | | |
| Cancer Stage = Local (n = 9,064) | | | | | | |
| No mammogram | 4,672 (72.4%) | 1,405 (89.9%) | *Reference* | 628 (76.7%) | 211 (91.3%) | *Reference* |
| Mammogram | 1,779 (27.6%) | 158 (10.1%) | **0.57 (0.48,0.68)** | 191 (23.3%) | 20 (8.7%) | - |
| Cancer Stage = Regional (n = 4,779) | | | | | | |
| No mammogram | 2,445 (81.2%) | 1,144 (91.3%) | *Reference* | - | - | *Reference* |
| Mammogram | 565 (18.8%) | 109 (8.7%) | **0.63 (0.51,0.76)** | - | - | - |
| Cancer Stage = Distant (n = 671) | | | | | | |
| No mammogram | 132 (83.0%) | 400 (93.9%) | *Reference* | - | - | *Reference* |
| Mammogram | 27 (17.0%) | 26 (6.1%) | **0.65 (0.43,0.98)** | - | - | - |
| Education Level | | | | | | |
| Some High School or Less (n = 1,405) | | | | | | |
| No mammogram | 356 (80.5%) | 702 (93.2%) | *Reference* | - | - | *Reference* |
| Mammogram | 86 (19.5%) | 51 (6.8%) | **0.62 (0.46,0.82)** | - | - | - |
| High School Degree (n = 3,383) | | | | | | |
| No mammogram | 1,488 (79.2%) | 1,014 (92.0%) | *Reference* | - | - | *Reference* |
| Mammogram | 391 (20.8%) | 88 (8.0%) | **0.53 (0.42,0.66)** | - | - | - |
| Some College (n = 3,202) | | | | | | |
| No mammogram | 1,552 (74.5%) | 694 (89.7%) | *Reference* | - | - | *Reference* |
| Mammogram | 531 (25.5%) | 80 (10.3%) | **0.47 (0.37,0.60)** | - | - | - |
| College Degree (n = 1,458) | | | | | | |
| No mammogram | 792 (72.7%) | 204 (87.2%) | *Reference* | - | - | *Reference* |
| Mammogram | 297 (27.3%) | 30 (12.8%) | **0.51 (0.35,0.76)** | - | - | - |
| Post College (n = 1,028) | | | | | | |
| No mammogram | 550 (69.1%) | 132 (88.6%) | *Reference* | - | - | *Reference* |
| Mammogram | 246 (30.9%) | 17 (11.4%) | **0.34 (0.20,0.57)** | - | - | - |
| Diagnosis Age | | | | | | |
| Diagnosis Age 40-50 (n = 2,615) | | | | | | |
| No mammogram | 1,680 (81.8%) | 266 (93.3%) | *Reference* | - | - | *Reference* |
| Mammogram | 374 (18.2%) | 19 (6.7%) | 0.98 (0.78,1.22) | - | - | - |
| Diagnosis Age 51-74 (n = 9,107) | | | | | | |
| No mammogram | 4,838 (74.1%) | 1,397 (90.5%) | *Reference* | 635 (78.1%) | 207 (93.7%) | *Reference* |
| Mammogram | 1,691 (25.9%) | 147 (9.5%) | **0.63 (0.42,0.96)** | 178 (21.9%) | 14 (6.3%) | - |
| Diagnosis Age 75+ (n = 2,792) | | | | | | |
| No mammogram | 731 (70.5%) | 1,286 (91.0%) | *Reference* | 94 (71.2%) | 197 (93.8%) | *Reference* |
| Mammogram | 306 (29.5%) | 127 (9.0%) | **0.49 (0.41,0.57)** | 38 (28.8%) | 13 (6.2%) | - |
| Imputed BMI | | | | | | |
| Underweight (n = 235) | | | | | | |
| No mammogram | - | - | *Reference* | - | - | *Reference* |
| Mammogram | - | - | **0.29 (0.09,0.95)** | - | - | - |
| Normal Weight (n = 5,977) | | | | | | |
| No mammogram | 3,094 (75.5%) | 1,109 (91.9%) | *Reference* | 371 (74.5%) | 156 (90.7%) | *Reference* |
| Mammogram | 1,006 (24.5%) | 98 (8.1%) | **0.50 (0.40,0.61)** | 127 (25.5%) | 16 (9.3%) | - |
| Overweight (n = 4,781) | | | | | | |
| No mammogram | 2,340 (75.3%) | 1,040 (89.7%) | *Reference* | - | - | *Reference* |
| Mammogram | 767 (24.7%) | 120 (10.3%) | **0.66 (0.55,0.80)** | - | - | - |
| Obese (n = 3,521) | | | | | | |
| No mammogram | 1,710 (75.4%) | 742 (91.2%) | *Reference* | - | - | *Reference* |
| Mammogram | 557 (24.6%) | 72 (8.9%) | **0.43 (0.34,0.56)** | - | - | - |
| *Adjusted on age, race, ethnicity, education, BMI, and CCI score, where appropriate. | | | | | | |

| **Supplemental Table 4: Adjusted odds ratio of breast cancer given a screening mammogram was administered between one and three years before index date, stratified by the index date year (n = 77,633)** | | | |
| --- | --- | --- | --- |
| **Years** | **Cases, n (%)** | **Controls, n (%)** | **Odds Ratio (95% CI)** |
| 1998-2002 | | | |
| Mammogram Performed in Timeframe: | | | |
| No mammogram | 1,296 (95.4%) | 3,856 (96.1%) | *Reference* |
| Mammogram | 62 (4.6%) | 157 (3.9%) | 1.11 (0.80,1.54) |
| Number of Mammograms Performed in Timeframe | | | |
| 0 | 1,296 (95.4%) | 3,856 (96.1%) | *Reference* |
| 1 | 39 (2.9%) | 115 (2.9%) | 0.86 (0.57,1.29) |
| 2 | 21 (1.6%) | 41 (1.0%) | **1.91 (1.06,3.44)** |
| 3+ | 2 (0.2%) | 1 (0.0%) | 4.27 (0.35,52.69 |
| 2003-2007 | | | |
| Mammogram Performed in Timeframe: | | | |
| No mammogram | 3,247 (93.8%) | 13,649 (95.1%) | *Reference* |
| Mammogram | 215 (6.2%) | 703 (4.9%) | **1.34 (1.13,1.58)** |
| Number of Mammograms Performed in Timeframe | | | |
| 0 | 3,247 (93.8%) | 13,649 (95.1%) | *Reference* |
| 1 | 128 (3.7%) | 437 (3.0%) | **1.26 (1.02,1.56)** |
| 2 | 85 (2.5%) | 266 (1.9%) | **1.43 (1.10,1.85)** |
| 3+ | 2 (0.1%) | 0 (0.0%) | N/A |
| 2008-2012 | | | |
| Mammogram Performed in Timeframe: | | | |
| No mammogram | 3,604 (80.0%) | 17,144 (82.3%) | *Reference* |
| Mammogram | 898 (20.0%) | 3,696 (17.7%) | **1.19 (1.09,1.30)** |
| Number of Mammograms Performed in Timeframe | | | |
| 0 | 3,604 (80.1%) | 17,144 (82.3%) | *Reference* |
| 1 | 534 (11.9%) | 2,539 (12.1%) | 1.03 (0.93,1.15) |
| 2 | 359 (8.0%) | 1,162 (5.6%) | **1.53 (1.34,1.75)** |
| 3+ | 5 (0.1%) | 5 (0.0%) | **4.39 (1.14,16.11)** |
| 2013-2017 | | | |
| Mammogram Performed in Timeframe: | | | |
| No mammogram | 3,412 (65.7%) | 16,209 (67.8%) | *Reference* |
| Mammogram | 1,782 (34.3%) | 7,703 (32.2%) | **1.11 (1.04,1.19)** |
| Number of Mammograms Performed in Timeframe | | | |
| 0 | 3,412 (65.7%) | 16,209 (67.8%) | *Reference* |
| 1 | 836 (16.1%) | 4,252 (17.8%) | 0.94 (0.86,1.02) |
| 2 | 750 (14.4%) | 2,876 (12.0%) | **1.29 (1.18,1.42)** |
| 3+ | 196 (3.8%) | 575 (2.4%) | **1.60 (1.33,1.93)** |
| * Adjusted on race, smoking status, first-degree family member with breast cancer history, ethnicity, education level, BMI, and CCI score. | | | |
